# Contour-Based Chain-Code Serialization for Lossless Compression of Voxelized 3D Objects

**DOI:** 10.3390/e28070774

**Published:** 2026-07-08

**Authors:** Esteban-Alejandro Durán-Yáñez, Mario-Alberto Rodríguez-Díaz, Ricardo Mendoza-González, Francisco-Javier Luna-Rosas, Julio-César Martínez-Romo

**Affiliations:** Departamento de Sistemas y Computación, Tecnológico Nacional de México/Instituto Tecnológico de Aguascalientes, Av. Adolfo López Mateos #1801 Ote., Fracc. Bona Gens, Aguascalientes 20255, Mexico; r21153080@aguascalientes.tecnm.mx (E.-A.D.-Y.); mendozagric@aguascalientes.tecnm.mx (R.M.-G.); fjluna@aguascalientes.tecnm.mx (F.-J.L.-R.); julio.mr@aguascalientes.tecnm.mx (J.-C.M.-R.)

**Keywords:** voxel compression, chain code, lossless compression, source serialization, contour representation, binary occupancy grid, octree, ModelNet40, G-PCC, entropy coding

## Abstract

Voxel representations provide a simple way to represent three-dimensional objects as binary occupancy signals, but dense voxel grids and direct sparse encodings remain costly at medium and high resolutions. This paper addresses the gap between conventional dense-grid, octree, and point-cloud-codec representations and deterministic contour-first source serialization for exact binary voxel occupancy. We propose a contour-based chain-code serialization that decomposes a voxel grid into two-dimensional slices, extracts foreground components and holes, encodes their contours using F4, 3OT, and F8 variants, and separates contour symbols from positional metadata before applying general-purpose lossless compression. The method is evaluated on 3983 ModelNet40-derived voxelized objects across 40 classes and resolutions N = 8, 16, 32, 64, 128, 256, and 512, using the X-axis for the main evaluation. It is compared against OCC1, BINVOX, breadth-first octree masks, and geometry-only G-PCC. The proposed streams are not competitive at N = 8, where zstd-compressed octree masks achieve the best mean bpv. From N = 16 onward, however, the best proposed stream outperforms the strongest evaluated baseline, with gains increasing from 20.93% at N = 16 to 84.37% at N = 512. The best proposed configuration is zstd + 3OT at N = 8 and N = 16, while zstd + F8 dominates from N = 32 through N = 512. Entropy, ablation, timing, memory, and validation analyses further show that the advantage comes from the interaction between contour-aware source serialization and backend compression, rather than from the backend compressor alone.

## 1. Introduction

Three-dimensional objects can be represented as polygon meshes, point clouds, signed distance fields, implicit functions, octrees, or binary voxel grids. Among these alternatives, voxel occupancy grids are conceptually simple: each spatial cell is either occupied or empty. This simplicity makes voxel grids attractive for geometric processing, learning-based shape analysis, and controlled experimental comparisons. However, explicit voxel occupancy representations also introduce substantial storage overhead, especially as spatial resolution increases. Even when only occupied voxels are stored, neighboring cells and slice-wise contours often exhibit regularities that plain coordinate lists or generic binary layouts do not exploit.

Lossless compression of voxelized objects is relevant whenever geometric occupancy must be preserved exactly. Examples include reproducible geometric benchmarks, scientific shape analysis, digital preservation of derived voxel data, and controlled comparisons between representations. Unlike lossy mesh simplification or point-cloud quantization, lossless voxel compression seeks to recover the exact original binary occupancy after decoding. The challenge is therefore not only to reduce file size, but also to define a representation that exposes redundancy to downstream entropy coders while retaining all information required for exact reconstruction.

In contrast to a literature dominated by dense-grid storage, sparse octree traversal, standardized point-cloud codecs, and increasingly learned occupancy-probability models, this work explores a contour-based approach to lossless compression of voxelized 3D objects. The central idea is to transform a three-dimensional occupancy grid into a structured sequence of two-dimensional contours, hole contours, and positional metadata before applying a backend lossless compressor. The contribution is therefore representation-level: the proposed serialization aims to expose geometric regularities that are less explicit in conventional voxel scans, coordinate lists, or generic sparse-tree traversals.

The proposed approach is motivated by classical chain coding of digital curves. Chain codes provide compact symbolic descriptions of contours by recording directional steps or relative transitions along a discrete boundary. In the present work, chain codes are not applied to isolated 2D shapes, but rather to the slice-wise structure induced by voxelized 3D objects. This makes the representation sensitive to both geometry and stream organization: compression efficiency depends on the contour coder, object ordering, hole handling, and the separation of positional metadata from the contour stream.

LiDAR and point-cloud completion pipelines provide an important adjacent context for this representation-level study. Recent work on Point-KAN, for example, addresses trustworthy real-time 3D point-cloud completion for 6G-IoT scenarios such as autonomous driving, AR/VR, UAV-based mapping, and industrial robotics [1]. Completion and compression play complementary roles in such pipelines: completion estimates missing geometry from partial or noisy scans, whereas lossless compression preserves a given voxel or occupancy representation exactly for storage, transmission, auditing, or reproducible downstream processing. The present work, therefore, should not be interpreted as a LiDAR completion method; rather, it studies exact source serialization for binary voxel occupancy, which can complement point-cloud processing workflows after voxelization or when completed geometry is converted into occupancy grids.

A methodological clarification is important. The goal of this paper is not primarily to determine whether one chain-code alphabet is universally superior. Instead, the main contribution is a contour-based source serialization for voxelized objects and an experimental comparison against representative voxel-compression baselines. F4, 3OT, and F8 serve as contour-coding instantiations of the same serialized framework, allowing the impact of contour connectivity, symbol alphabet, and stream organization to be evaluated under identical metadata and compression settings.

The remainder of the paper is organized as follows. Section 2 reviews relevant background on chain codes, voxel representations, octree-based encodings, point-cloud compression, and learned occupancy models. Section 3 presents the proposed contour-based serialization method with formal notation. Section 4 describes the experimental design, datasets, resolutions, methods, compressors, baselines, and reproducibility settings. Section 5 reports the revised 40-class compression results, entropy analysis, axis sensitivity, ablations, runtime, memory usage, and validation results. Section 6 discusses interpretation, limitations, and threats to validity. Section 7 concludes the paper and outlines future work.

The main contributions of the paper are:

- A lossless slice-wise contour-based serialization framework for binary voxel occupancy models, including explicit handling of foreground components, holes, and relative start-position metadata.
- A unified evaluation of F4, 3OT, and F8 as contour-coding instantiations of the same serialized representation.
- A 40-class ModelNet40-derived evaluation on 3983 voxelized objects along the X axis, comparing the proposed streams against OCC1, BINVOX, octree_mask_bfs, and geometry-only G-PCC.
- An empirical order-0, order-1, and order-2 entropy analysis showing that 3OT is strongest under order-0 ideal coding, while F8 dominates contextual order-1 and order-2 criteria.
- A four-class deep analysis with X/Y/Z axes, exact reconstruction checks, FULL/ABS/SEP/INT ablations, and encoding/decoding timing, memory, and GC measurements.
- A reproducibility package reporting software versions, gzip and zstd levels, G-PCC/TMC13 settings, obj2voxel/VoxelList-RGB details, and all final result workbooks.

## 2. Background and State of the Art

### 2.1. Source Coding, Chain Codes, and Contour-Aware Representation

Lossless compression of voxelized 3D objects can be formulated as a source-coding problem: the objective is to describe a binary occupancy signal with as few bits as possible while guaranteeing exact reconstruction. This view follows Shannon’s information-theoretic formulation of communication and data compression, where the statistical structure of a source determines how efficiently it can be represented [2]. In the present setting, the source is not a natural image or a continuous point set, but a finite binary volumetric signal whose occupied and empty cells must be recovered exactly.

A second foundation is Freeman’s chain-code representation of digital contours [3,4]. Chain codes replace unordered or rasterized boundary pixels with ordered directional symbols, making local geometric continuity explicit. Although classical chain coding is two-dimensional, it is highly relevant for voxelized 3D objects because a volume can be decomposed into 2D slices whose cross-sections contain contours, holes, and connected components. The main hypothesis explored in this work is therefore representation-level: before a backend entropy coder is applied, the 3D occupancy field can be serialized into a contour-aware symbol stream whose regularities are more favorable than those of dense grids, coordinate lists, or generic tree traversals.

Within this chain-code lineage, the three-orthogonal (3OT) code was introduced by Sánchez-Cruz and Rodríguez-Dagnino for bilevel image compression as a relative contour code based on a three-symbol alphabet [5]. A broader efficiency study later compared 3OT, F4, F8, VCC, and related chain codes for binary objects [6], providing a direct precedent for treating F4 and 3OT as alternative contour-coding instantiations. Related work by Sánchez-Cruz et al. extended relative chain coding to three-dimensional discrete curves, showing that relative symbolization is also meaningful in voxel-based 3D settings [7].

More specifically, chain-code compression has also been studied directly for two-dimensional binary and rasterized images. Žalik and collaborators proposed lossless compression of chain-code streams using a move-to-front transform and adaptive run-length encoding [8], later generalized this line with a universal chain-code compression method [9], and more recently contributed to efficient compressed storage and fast reconstruction of large binary images using chain codes [10]. These methods focus on compressing contour-symbol streams or chain-coded binary masks, whereas the present work embeds the same 2D contour-stream principle into a three-dimensional, slice-wise voxel-occupancy serialization with explicit foreground contours, hole contours, and positional metadata.

### 2.2. Binary Voxel-Surface Coding

The most directly related classical literature is binary voxel-surface compression. Kim and Lee proposed a lossless pattern-code representation for 3D binary voxel surfaces, using local 3 × 3 × 3 neighborhoods to characterize surface configurations and exploit the correlation between adjacent surface voxels [11]. Kwon et al. later introduced progressive encoding of binary voxel models using pyramidal decomposition, boundary partitioning, and context-adaptive arithmetic coding [12]. These works are important because they recognized that geometric redundancy is concentrated near surfaces and boundaries rather than being uniformly distributed throughout the volume.

The proposed method differs from these voxel-surface coders in two ways. First, it operates slice-by-slice and represents each slice component using chain-code contours, rather than assigning local 3D pattern codes to surface voxels. Second, it separates contour symbols from positional metadata and then evaluates how generic entropy compressors exploit the resulting stream. Thus, the contribution is not a new arithmetic coder for voxel events, but rather a deterministic source representation designed to expose contour continuity in binary occupancy data.

More recently, Repnik et al. proposed an algorithm for representing voxelized solids using chain codes, storing the resulting sequences in a compact binary format and reporting lower entropy and improved gzip/bzip2 compressibility for their token streams [13]. That work is closely related at the level of voxelized-surface representation. The present paper differs by using a slice-wise 2D contour serialization with explicit hole and metadata streams and by benchmarking lossless occupancy compression over a 40-category ModelNet40-derived set against BINVOX, SVO/octree, OCC1, and G-PCC baselines.

### 2.3. Octree Representations and Standardized Point-Cloud Compression

Octree-based representations are the dominant conventional baseline for sparse 3D geometry. Schnabel and Klein presented an octree-based point-cloud compression method that became an important bridge between sparse voxel structures and later point-cloud compression pipelines [14]. Sparse voxel octrees further exploit the fact that many subregions of a 3D grid are empty, replacing dense volumetric storage with recursive occupancy decisions [15]. These methods are strong baselines because recursive subdivision suppresses empty space and turns geometry into compact occupancy symbols.

Modern point-cloud compression has been strongly shaped by MPEG standardization. The G-PCC family, formalized in ISO/IEC 23090-9, provides a mature geometry-based reference framework for point-cloud compression [16,17]. In this study, G-PCC is used as an adjacent and demanding baseline because occupied voxel centers can be interpreted as a point cloud. Nevertheless, the comparison is not perfectly symmetric: G-PCC is designed for general point-cloud geometry, whereas the proposed method is specialized for exact binary voxel occupancy and contour structure.

### 2.4. Learned Occupancy Models and Recent PCC Trends

Recent research has shifted from handcrafted voxel or contour coders toward learned probability models over octree or sparse-voxel events. OctSqueeze introduced an octree-structured entropy model for LiDAR point-cloud compression [18]. VoxelContext-Net combined octree organization with local voxel context to improve entropy modeling for static and dynamic point clouds [19]. VoxelDNN and MSVoxelDNN used learned voxel-context models for lossless point-cloud geometry, with MSVoxelDNN reducing sequential autoregressive decoding by grouping voxels in a multiscale order [20,21]. SparsePCGC extended this trend using multiscale sparse tensor processing for point-cloud geometry compression [22], while Nguyen and Kaup learned conditional probability models for joint lossless geometry and attribute compression [23].

These learned methods are highly relevant to the state of the art, but they address a different layer of the compression problem. Most of them improve the probability model or entropy coder over octree, voxel-block, or sparse-tensor events. By contrast, this paper examines whether a deterministic contour-first serialization can improve the source stream before backend compression. The proposed method should therefore be interpreted as complementary to learned entropy modeling: in principle, a contour-based stream could be paired with stronger statistical or neural coders in future work.

### 2.5. Metrics, Datasets, and Positioning of This Work

Evaluation protocols differ between lossless occupancy compression and lossy point-cloud compression. Lossy PCC studies commonly report bitrate together with distortion measures such as point-to-point and point-to-plane PSNR, and sometimes BD-rate; point-to-plane distortion was introduced to better reflect local surface fidelity than pure nearest-neighbor distances in several compression settings [24]. In this paper, however, reconstruction is exact, so D1/D2 PSNR and BD-rate are not primary metrics. The appropriate measures are compressed size, compression ratio, bits per voxel, and bits per occupied voxel under lossless reconstruction.

The dataset context is also different. ModelNet40 is a canonical object-centric CAD benchmark for 3D shape analysis [25], whereas many recent PCC studies evaluate on dynamic human captures, LiDAR sequences, or large scene datasets. The present experiments are therefore best described as an evaluation of lossless compression for voxelized CAD-like object occupancy, with G-PCC and octree methods serving as strong adjacent baselines. This framing avoids overstating the claim as a universal point-cloud codec and instead positions the paper as a representation-level study for binary voxel objects.

Taken together, these observations suggest a focused research gap: the literature contains strong octree baselines and increasingly powerful learned occupancy models, but comparatively little recent work revisits deterministic contour-first serialization for exact voxel occupancy. The present manuscript addresses this gap by combining a classical chain-code view of contours with an evaluation against dense-grid, coordinate-list, octree/SVO, and G-PCC-style representations under gzip and zstd compression. Table 1 summarizes the representative works that define this context, and Table 2 places the main developments on a chronological timeline.

## 3. Proposed Contour-Based Serialized Representation

**Figure 1:**
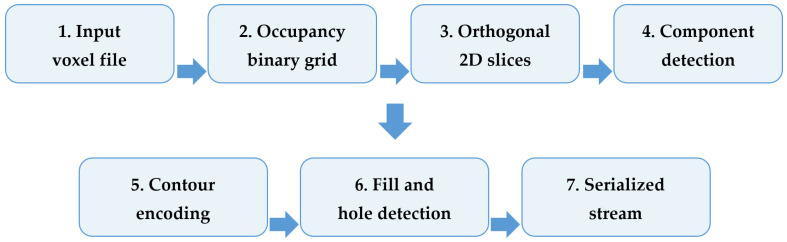
Pipeline of the proposed contour-based representation for voxelized 3D objects.

This section describes the proposed lossless representation for voxelized 3D objects. The method operates slice-by-slice, converting a binary occupancy volume into a serialized stream of contour codes and positional metadata. Figure 1 summarizes the processing pipeline.

### 3.1. Input Voxel Model and Binary Occupancy Grid

Let VoxelList-RGB denote the intermediate source voxel-list format used by the implementation. In the experimental pipeline, VoxelList-RGB files are generated from Wavefront OBJ meshes using the obj2voxel command-line voxelizer [26] at the selected target resolution N. The term VoxelList-RGB is used in this manuscript to describe the resulting coordinate-color voxel list, rather than to introduce an additional compression baseline. Formally, VoxelList-RGB is the finite ordered list *L*_RGB_ = ((*x_i_*, *y_i_*, *z_i_*, *r_i_*, *g_i_*, *b_i_*))*i = 1^n^*, where (*x_i_*, *y_i_*, *z_i_*) ∈ ℤ^3^ are voxel coordinates and (*r_i_*, *g_i_*, *b_i_*) ∈ {0, …, 255}^3^ are RGB attributes. The evaluated lossless representation uses only the geometric projection of this list; color is read during loading but discarded before occupancy coding. The occupied voxel set is written as

$$V=\left\{\left(x_{i},\;y_{i},\;z_{i}\right)\;:\;\left(x_{i},\;y_{i},\;z_{i},\;r_{i},\;g_{i},\;b_{i}\right)\;\in \;L_{RGB}\right\}.$$

The voxel list is converted into a binary occupancy grid M, where occupied positions are foreground and all other positions are background:

$$M\left(x,\;y,\;z\right)\;=\;\left\{\begin{array}{c} 1\;\mathrm{if}\;\left(x,\;y,\;z\right)\;\in \;V\\ 0\;\mathrm{otherwise} \end{array}\right.$$

The resulting source object is therefore a binary volumetric signal. Exact reconstruction requires recovering M without changing any occupied or empty cells.

### 3.2. Slice Extraction

The volume is decomposed into a sequence of two-dimensional slices. In the default configuration, the implementation processes the grid along the X axis, so the *k*-th slice is a binary YZ image. We use (*u*, *v*) to denote the in-slice coordinates, with *u* along the Y axis and *v* along the Z axis, and write:

$$S_{k}\left(u,v\right)=M\left(k,u,v\right),k=1,\ldots ,K.$$

The method can be generalized to other axes, but the single-axis formulation is sufficient to define the representation evaluated here. Each slice is processed independently as a binary image, and the full model is reconstructed by decoding all slices in order.

### 3.3. Connected-Component Detection and Initial Position

Each slice is scanned in raster order to locate the first foreground pixel that has not been processed and has not been labeled as a hole. This pixel becomes the starting seed of the next component to be traced. The effective contour-continuation rule is coder-dependent: F4 uses a four-neighbor orthogonal pixel-center traversal, 3OT uses a pixel-center relative-transition rule with diagonal inspection, and F8 permits eight-direction pixel-center continuation. Holes are identified during internal reconstruction (Section 3.5) as background pixels enclosed by an already-traced foreground contour. For a slice of width W and height *H*, the starting pixel of the *j*-th object is denoted by *p_j_* = (*u_j_*, *v_j_*), where the slice axes (*u*, *v*) follow the convention introduced in Section 3.2. Its linearized position is

$$l_{j}=v_{j}\;W+u_{j}.$$

The linearized position is not stored repeatedly as an absolute coordinate. Instead, it is later converted into a relative offset with respect to the previous object in the same ordered stream group.

### 3.4. Contour Encoding of Foreground Objects

Once a component seed has been detected, its boundary is traced from the starting pixel and converted into a chain-code sequence. All evaluated tracers operate on integer pixel-center coordinates of the slice grid, rather than on cracks or sub-pixel edges. The starting pixel is the first foreground pixel encountered in a row-major scan of the slice, and the initial direction *d* is fixed to + *u* (right) by convention. The traversal rule is specific to each chain-code instantiation, as detailed below. The code of the *j*-th *L_j_* object in a slice is denoted by

$$C_{j}=\left(c_{j,1},c_{j,2},\ldots ,c_{j,L_{j}}\right),$$

where *L_j_* is the number of emitted symbols, and the alphabet depends on the contour coder. In the F4 variant [3,4,6], the alphabet is {0, 1, 2, 3}, where each symbol denotes one of four orthogonal moves on the slice grid: 0 = right (+*u*), 1 = up (+*v*), 2 = left (−*u*), and 3 = down (−*v*). F4 uses a deterministic left-priority rule relative to the current direction d: the candidate directions are examined as *d* + 90 (turn left), *d* (continue straight), *d* − 90 (turn right), and *d* + 180 (reverse), with angular offsets expressed in degrees. The first foreground neighbor encountered in this order becomes the next boundary pixel, and *d* is updated to the selected direction.

In the 3OT variant [5,6], the alphabet is {0, 1, 2}, where each symbol denotes a relative transition with respect to the previous direction: 0 = continue straight, 1 = turn left, and 2 = turn right. The evaluated 3OT tracer is pixel-centered and is not a crack-code variant. At each step, it examines the foreground at the current direction d and at d + 45. If foreground is present in both, the tracer emits a turn symbol and advances diagonally by one pixel center along d + 45. If foreground is present at d but not at d + 45, it emits 0 and advances orthogonally along d. If foreground is absent at d, it emits a turn symbol and rotates the current direction by −90 degrees without advancing the position. This rotate-without-advance branch is one source of longer 3OT streams relative to F4 in concave configurations, as discussed in Section 5.3.

In the F8 variant, the emitted symbols describe absolute eight-direction transitions: 0 = right, 1 = upper-right, 2 = up, 3 = upper-left, 4 = left, 5 = lower-left, 6 = down, and 7 = lower-right. F8 uses a left-priority eight-direction crawler that generalizes the F4 traversal rule. At each step, the candidate directions are examined relative to the current direction *d* as *d* + 90, *d* + 45, *d*, *d* − 45, *d* − 90, *d* − 135, *d* + 180, and *d* + 135, with angular offsets expressed in degrees. The first foreground neighbor encountered becomes the next boundary pixel, and d is updated to the selected direction. This evaluated F8 tracer is an implementation-specific left-priority eight-direction crawler and should not be interpreted as a general evaluation of all possible eight-neighbor contour-tracing algorithms.

For non-degenerate contours, object termination is implicit: decoding proceeds until the reconstructed contour returns to the initial pixel under the corresponding local continuation condition. Therefore, no explicit end marker is required between consecutive component codes.

Degenerate one-pixel components are handled explicitly using a reserved initial-symbol convention. A single isolated pixel admits no boundary walk, so each tracer emits a one-symbol codeword that cannot occur as the first symbol of a valid non-degenerate contour under the same raster-order initialization and traversal convention: 2 for F4, 1 for 3OT, and 4 for F8. This reservation applies only at the initial position of a component code; in all other positions, the same symbol values retain their ordinary F4, 3OT, or F8 meanings.

For non-degenerate components, this implicit termination rule is central to the serialized layout. Instead of inserting a separator after every object, the decoder uses contour closure to decide where one object ends, and the next begins. During decoding, an initial 2 in F4, an initial 1 in 3OT, or an initial 4 in F8 is interpreted as the reserved isolated-component codeword for the active contour coder; the decoder then reconstructs a single occupied pixel at the stored start position and advances to the next component. Otherwise, the contour is decoded normally until closure. In either case, the next unread symbol is then interpreted as the first symbol of the following object.

### 3.5. Internal Reconstruction, Filling, and Hole Detection

After a foreground contour has been encoded, it is immediately decoded in an auxiliary binary matrix. This internal reconstruction is not an output format; it is a bookkeeping mechanism used to recover the filled region, update the processed mask, and identify holes. First, an external flood fill is applied to an expanded matrix with a one-pixel border. This marks the outside background and prevents exterior empty space from being confused with enclosed cavities. Second, points surrounded by the reconstructed boundary are filled. Third, the filled reconstruction is compared with the original source slice. Pixels inside the filled region that are background in the original slice are labeled as holes.

Explicit hole handling is necessary because an outer contour alone does not fully describe a multiply connected slice component. Without hole encoding, internal cavities would be lost during reconstruction. The proposed method, therefore, treats holes as a second category of objects to be encoded after foreground components.

### 3.6. Foreground and Hole Object Sets

For each slice s, the encoder produces an ordered foreground set and an ordered hole set, where each *f*_*s*,*i*_ (respectively *h*_*s*,*i*_) denotes the chain-code sequence of the *i*-th foreground component (respectively *i*-th hole component) in slice *s*, as defined in Section 3.4:

$$F_{s}=\left\{f_{s,1},f_{s,2},\ldots ,f_{s,n_{s}}\right\},H_{s}=\left\{h_{s,1},h_{s,2},\ldots ,h_{s,m_{s}}\right\}.$$

Foreground components are processed first. Hole components are then inverted to foreground and encoded using the same contour-coding procedure. This foreground-first, hole-second ordering is part of the representation definition and is preserved in the final stream.

### 3.7. Relative Positional Metadata

For each ordered stream group, absolute starting positions are converted into relative offsets. If ℓ_j is the linearized starting position of the *j*-th object, the stored distance is

$$d_{j}=l_{j}-l_{j-1},with\;l_{0}=0.$$

This relative representation reduces positional overhead and creates integer sequences that are more regular than repeated absolute coordinate pairs. Distances are stored separately from contour symbols to preserve the internal regularity of each stream component.

### 3.8. Serialized Stream Organization

At the model level, all foreground contour codes are concatenated first, followed by all hole contour codes and a metadata suffix. The stream is represented as

$$S=C_{F}∥C_{H}∥M_{D},$$

where *C_F_* is the concatenation of foreground contour codes, *C_H_* is the concatenation of hole contour codes, and *M_D_* is the metadata suffix. The metadata suffix begins with the first hyphen character followed by slice dimensions, and subsequent hyphens delimit slice-level distance lists:

$$M_{D}=‘-’W,H∥D_{F}∥D_{H}.$$

**Figure 2:**
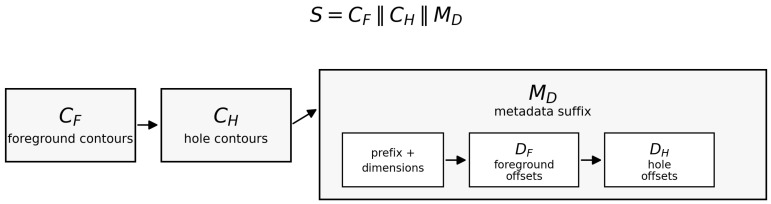
Organization of the serialized stream *S* = *C_F_* || *C_H_* || *M_D_*. The metadata suffix M_D contains the prefix delimiter, slice dimensions, and the ordered relative-offset lists *D_F_* and *D_H_* for foreground and hole components.

The first hyphen has a different semantic role from later hyphens: it introduces metadata and dimensions. All subsequent hyphens separate per-slice distance lists. Figure 2 shows the stream organization.

Because the contour alphabets do not use the hyphen character, the first hyphen in the serialized file provides an unambiguous delimiter between contour symbols and metadata. The metadata block stores the slice dimensions and the ordered relative starting positions for foreground components and holes. Therefore, during decoding, the number and order of components to be reconstructed in each slice are obtained from the metadata lists, while the end of each individual contour is determined by the contour-closure condition.

### 3.9. Decoding and Lossless Reconstruction

Decoding reverses the serialization procedure. The decoder reads the metadata to recover slice dimensions and relative start positions. For each object, the starting position is reconstructed. For non-degenerate objects, the contour stream is decoded symbol by symbol until the boundary returns to the starting pixel. Degenerate one-pixel components are decoded using the reserved initial-symbol rule described in Section 3.4. The interior is filled; holes are subtracted according to the hole stream, and all decoded slices are stacked to recover M. Because the representation stores contour geometry, start-position metadata, explicit hole information, and the isolated-component convention, the reconstruction is lossless under a correct decoder.

### 3.10. Role of F4, 3OT, and F8

F4, 3OT, and F8 are treated as contour-coding instantiations within the same serialized representation. F4 records absolute orthogonal moves produced by a four-direction left-priority pixel-center crawler. 3OT records relative transitions produced by a pixel-center tracer that combines orthogonal advances, diagonal advances, and rotate-without-advance events. F8 records absolute eight-direction symbols produced by the evaluated left-priority eight-direction crawler. The global layout, metadata suffix, delimiter convention, and slice ordering remain the same. Therefore, differences among F4, 3OT, and F8 are attributable to the emitted contour-symbol stream rather than to changes in the overall file organization. The final results show that 3OT is competitive at coarse resolutions, while F8 better exposes contextual and sequential structure at medium and high resolutions under zstd compression.

## 4. Experimental Design

### 4.1. Dataset and Voxelization Scope

The target benchmark is ModelNet40, a widely used 3D CAD dataset containing 40 object categories. The final result set contains 3983 voxelized objects: 38 classes contain 100 objects, bowl contains 84 objects, and cup contains 99 objects. The main compression evaluation uses all 40 classes along the X-axis. A complementary deep analysis is performed on the airplane, bookshelf, chair, and vase using the X, Y, and Z axes for exact reconstruction, timing, and ablation analysis.

The resolutions considered by the pipeline are 8, 16, 32, 64, 128, 256, and 512. For each object and resolution, the source model is voxelized, converted into the required input format, encoded with the proposed methods, and compared against baseline representations. The 40-class X-axis evaluation is used for the main compression claims, while the four-class X/Y/Z analysis is used to address sensitivity, exactness, and resource-use questions.

### 4.2. Compared Representations and Baselines

The experimental comparison separates representation from the compressor. The Method field denotes the source representation, whereas the Compressor field denotes the external lossless compressor applied to that representation. Table 3 summarizes the final representation-level methods and baselines. The custom_svo_leaf2 representation was generated during experimentation but is excluded from the final analysis because it is redundant with octree_mask_bfs. The octree-based baseline used throughout this paper, named octree_mask_bfs in formal listings (Table 3) and referred to in subsequent tables and figures as Octree BFS for brevity, is a pointerless sparse voxel octree in which occupancy is serialized as a breadth-first sequence of node masks rather than as explicit child pointers.

### 4.3. Compressors and G-PCC Settings

For stream-based methods, external lossless compressors are applied consistently. Results report gzip/DEFLATE [27] at level 9 and zstd [28] at level 19 for BINVOX [29], OCC1, octree_mask_bfs, chaincode_F4_text, chaincode_3OT_text, and chaincode_F8_text. G-PCC is evaluated separately through TMC13 [30] in geometry-only mode after converting occupied voxels to point coordinates. The command used for G-PCC is: tmc3.exe --mode=0 --uncompressedDataPath=<input.ply> --compressedStreamPath=<output.gpcc> --disableAttributeCoding=1.

### 4.4. Metrics

Let *B_m_* be the size in bytes of method *m* after optional compression, *B*_ref_ be the size of the selected reference representation, |*Ω*| be the total number of cells in the voxel grid, and | *V* | be the number of occupied voxels. The reported metrics are:

$${CR}_{m}=\frac{B_{ref}}{B_{m}}.$$

$${bpv}_{m}=\frac{8B_{m}}{\left|\Omega \right|}.$$

$${bpo}_{m}=\frac{8B_{m}}{\left|V\right|}.$$

The reported statistics include per-resolution means, standard deviations, 95% confidence intervals, class-wise rankings, and wins/losses comparing the best chain-code method against the best non-chain-code baseline. The principal publication metric is bits per voxel (bpv), because it normalizes compressed size by the full voxel grid and allows comparisons across resolutions.

### 4.5. Reproducibility and Implementation Notes

All experiments use the same voxelized occupancy source for all compared representations at each object-resolution pair. After voxelization, the encoding pipeline is deterministic for a fixed input object, target resolution, slicing axis, contour coder, serialization rule, and compressor setting. Each result record stores the dataset object, class, resolution, representation format, compressor, compressed bytes, voxel-grid size, occupied voxel count, bpv, bpo, and compression ratio.

The Supplementary Materials make the numerical evaluation auditable. The final package contains the 40-class X-axis compression workbook, the filtered compression record CSV with occupied-voxel counts filled, the entropy workbook for F4/3OT/F8, the four-class X/Y/Z ablation workbook, the encoding/decoding timing and validation workbook, the paired Wilcoxon bpv statistics package, the source-representation/backend-compressor analysis package, and a reproducibility protocol reporting Python v3.12 environment, gzip, zstd, obj2voxel, and TMC13/G-PCC settings.

The TMC13 executable used for G-PCC was tmc3 78ca512 + dirty, built for Windows x64 Release with Visual Studio 17 2022 and MSVC v143. The geometry-only command does not use a configuration file and disables attribute coding. VoxelList-RGB files are generated from OBJ files with Obj2Voxel.exe using the selected -r N resolution; the file contains x, y, z, and argb fields, but the present evaluation uses only binary geometric occupancy. Timing values for the chain-code encoder and decoder were measured on a conventional computing system, specifically a Windows 10 Home machine equipped with an Intel Core i7-10870H CPU @ 2.20 GHz and 32 GB RAM. This configuration corresponds to a commodity laptop-class workstation rather than specialized HPC infrastructure, so the reported runtimes provide a practical estimate of computational cost under realistic computing conditions. The remaining large-scale compression experiments were executed incrementally across multiple machines.

No training stage, stochastic model fitting, or randomized sampling is used by the proposed representation after voxelization. Consequently, remaining reproducibility sensitivity is mainly associated with software versions, command-line options, voxelization settings, and the availability of the derived intermediate files.

## 5. Results

This section reports the revised experimental results. Unless otherwise stated, lower bpv values indicate better compression. The main proposed-versus-baseline comparisons use the 40-category ModelNet40-derived X-axis evaluation with n = 3983 voxelized objects per resolution. Four-class X/Y/Z experiments are used for ablations, reconstruction checks, and computational-cost analysis.

### 5.1. Main 40-Class Compression Results

Table 4 summarizes the strongest proposed method and the strongest non-chain-code baseline at each resolution. The proposed representation is not uniformly best at the coarsest resolution. At N = 8, zstd + Octree BFS obtains the lowest mean bpv, and the best proposed stream, zstd + 3OT, is 22.17% larger. From N = 16 onward, however, the best proposed stream achieves lower mean bpv than the best evaluated baseline. The relative gain increases from 20.93% at N = 16 to 84.37% at N = 512.

**Figure 3:**
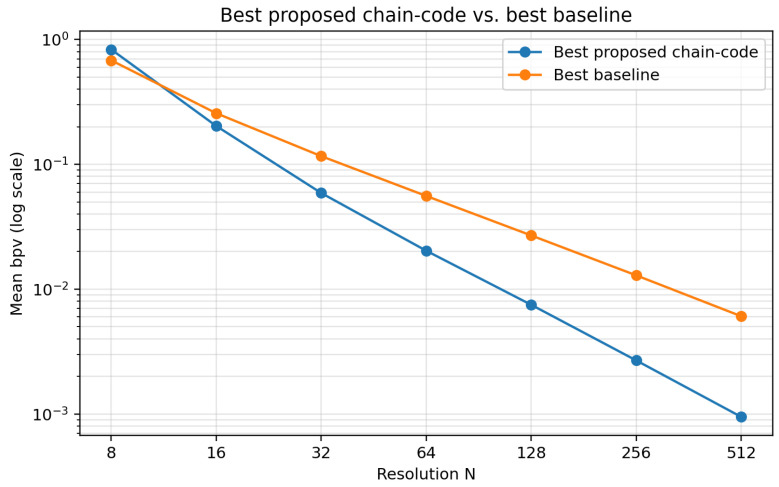
Mean bpv of the best proposed chain-code stream versus the best baseline by resolution.

**Figure 4:**
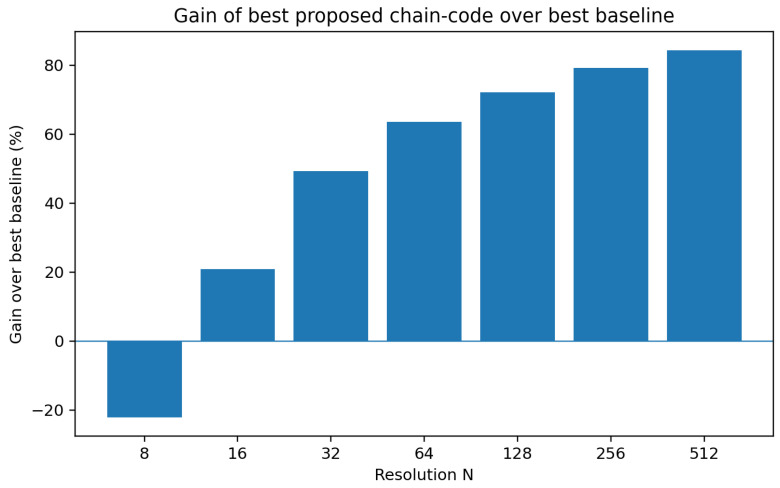
Percentage gain of the best proposed chain-code stream over the best baseline. Positive values favor the proposed method.

Figure 3 visualizes the same trend on a logarithmic bpv scale, while Figure 4 reports the percentage gain relative to the best baseline. The revised result changes the interpretation of the proposed variants: zstd + 3OT is best among the proposed streams at N = 8 and N = 16, whereas zstd + F8 dominates from N = 32 through N = 512.

To test whether the main bpv differences in Table 4 are consistent at the object level, paired Wilcoxon signed-rank tests were applied to the per-object bpv measurements. For each resolution, the best proposed configuration was compared against the strongest non-chain-code baseline reported in Table 4 using 3983 paired observations. The difference was defined as baseline bpv minus proposed bpv, so positive values favor the proposed method and negative values favor the baseline, see Table 5.

The test confirms the resolution-dependent behavior observed in the mean-bpv results. At N = 8, the strongest baseline remains significantly better than the best proposed stream. From N = 16 onward, the best proposed configuration is significantly better than the strongest evaluated baseline, and from N = 32 through N = 512, this advantage is obtained by zstd + F8.

### 5.2. Class-Wise Robustness

Class-wise wins/losses were computed by comparing the best available proposed stream against the best non-chain-code baseline within each ModelNet40 class and resolution. Table 6 shows that N = 8 is unfavorable to the proposed representation: only 3 of 40 classes are won by the chain-code stream. The behavior changes at N = 16, where 30 of 40 classes are won, and becomes increasingly consistent at higher resolutions, reaching 40 of 40 wins at N = 512.

**Figure 5:**
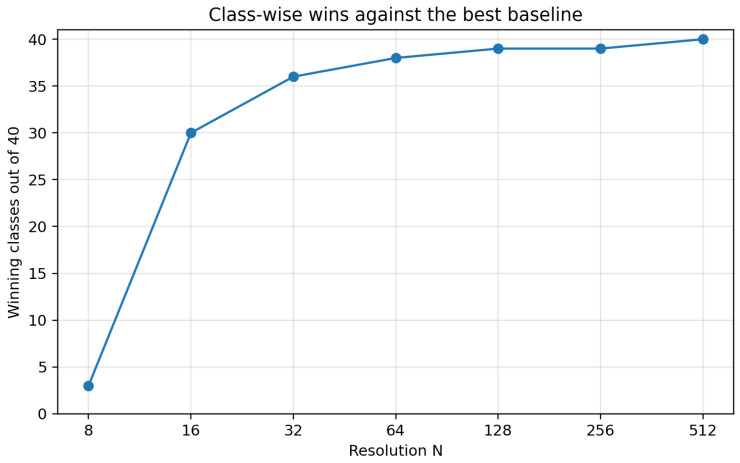
Number of ModelNet40 classes won by the best proposed chain-code stream against the best non-chain-code baseline across resolutions.

Figure 5 plots the number of winning classes across resolutions. This figure should be interpreted together with Table 4: the proposed method is worse on average and in most classes at N = 8, but becomes dominant across classes from N = 16 onward.

### 5.3. Entropy Analysis of F4, 3OT, and F8

To separate source-stream structure from backend-compressor behavior, empirical order-0, order-1, and order-2 ideal code-length estimates were computed for F4, 3OT, and F8 contour-symbol streams over the 40-class X-axis evaluation. Table 7 summarizes the resulting winner counts by criterion.

**Figure 6:**
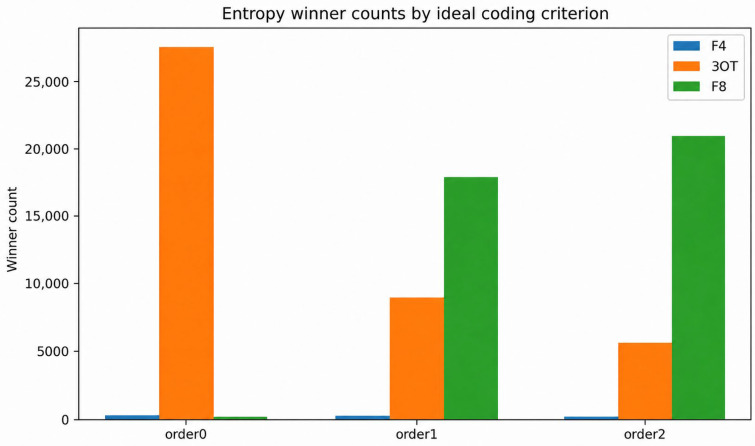
Entropy winner counts by ideal coding criterion.

3OT dominates the order-0 ideal code-length criterion, consistent with its compact alphabet. However, F8 dominates the order-1 and order-2 contextual criteria, indicating that it better exposes sequential and contextual regularities that are useful to dictionary-backed compressors such as zstd. Figure 6 shows these winner counts graphically.

The stream-length difference between F4 and 3OT should not be interpreted as a crack-code versus pixel-center effect. Both evaluated tracers operate on pixel centers. Instead, the difference arises from the dynamics of the 3OT relative-transition rule. At concavities, 3OT may emit a turn symbol while rotating the current direction without advancing the position, increasing the number of emitted symbols. Conversely, in local diagonal configurations, one 3OT symbol can advance diagonally by one pixel center. The empirical behavior, therefore, reflects the net balance between rotate-without-advance events, relative turns, and diagonal advances over the evaluated objects.

### 5.4. Ablation Study

Ablations were computed on the four-class deep-analysis subset, using airplane, bookshelf, chair, and vase across X/Y/Z axes and all seven resolutions. For each base contour coder, four serialization variants were evaluated. FULL denotes the complete proposed representation, including relative start-position metadata, implicit contour termination by closure, foreground-first/hole-second stream ordering, and explicit hole-contour encoding. ABS replaces relative start-position offsets with absolute linearized starting positions, isolating the effect of relative positional metadata. SEP inserts explicit separators between consecutive component codes, testing the cost of replacing implicit closure-based termination with explicit delimiters. INT interleaves foreground and hole components instead of storing all foreground contours before all hole contours, thereby evaluating the effect of the proposed foreground-hole ordering. Table 8 reports the zstd results for these variants. Positive overhead indicates that the ablated stream is larger than the corresponding FULL stream.

**Figure 7:**
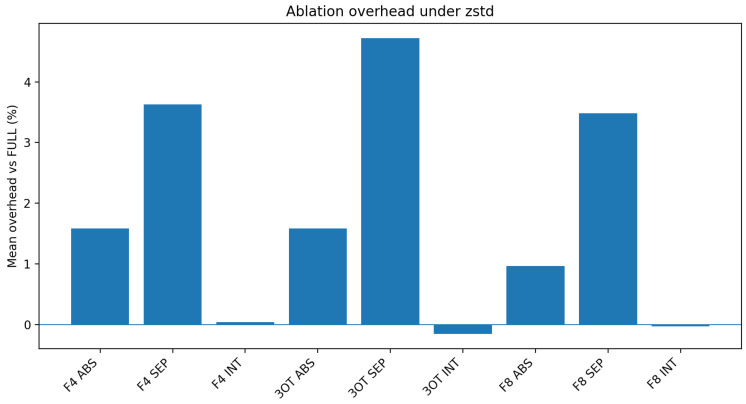
Ablation overhead under zstd relative to the FULL variant.

The SEP variant introduces the largest overhead for all three base codes, indicating that explicit separators are harmful once contour closure and metadata ordering are available. ABS also increases size, showing the benefit of relative positional metadata. INT has near-zero or slightly negative overhead in the aggregate, suggesting that foreground-hole interleaving is not consistently detrimental but does not replace the full representation. Figure 7 summarizes these overheads.

### 5.5. Encoding, Decoding, Memory, and Exact Reconstruction

Runtime and resource measurements were obtained for the proposed chain-code encoding and decoding stages. Table 9 reports per-resolution wall-clock times with 95% confidence intervals. Table 10 summarizes aggregate timing and memory values across X-axis resolutions.

**Figure 8:**
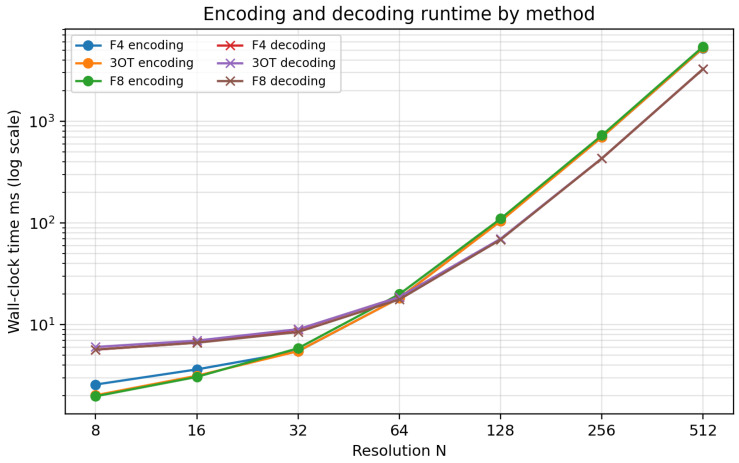
Encoding and decoding runtime by method and resolution.

Encoding and decoding times increase with resolution, as expected from the larger number of contour symbols and occupied structures. Differences among F4, 3OT, and F8 are modest compared with the effect of resolution. Figure 8 visualizes the encoding and decoding trends on a logarithmic time scale.

Table 11 summarizes the coverage of the result set and the exact-decoding validation records across the evaluated axes and methods. The table reports the total number of encoding and decoding records, the number of exact decoding records, and any flagged exceptions encountered during validation, providing a single auditable summary of the lossless guarantee of the proposed pipeline within the evaluated scope.

Across the X/Y/Z validation dataset, all 250,929 decoding records are exact. During this revision, seven Z-axis records were initially flagged as inexact and were traced to a guard in the slice-processing implementation that limited the recursive handling of nested foreground/hole structures to a fixed maximum of ten nesting levels; slice content nested beyond this limit was silently omitted at encoding time, so the defect was an encoding omission rather than a decoder failure, and the lossless property of the representation itself (Section 3.9) was not involved. The affected records correspond to two flower_pot objects at N = 256 and N = 512 sliced along the Z axis, whose horizontal cross-sections contain deeply nested concentric structures; the omitted content is exactly the innermost nested components, and the effect is connectivity-dependent: F4 and 3OT omitted identical voxel counts on the same objects, whereas F8, whose eight-direction continuation encloses additional diagonally bridged regions, omitted more voxels and exceeded the limit in one additional case. The guard was replaced with an iterative traversal without a fixed depth limit; nesting depth is, in any case, bounded by the slice geometry, so no practical limit is required, and the validation pipeline verifies exact reconstruction and complete symbol-stream consumption for every record. After this correction, the affected class was fully re-encoded and re-validated, covering 6300 records over 100 objects, seven resolutions, three contour coders, and three axes, and all 250,929 validation records now decode exactly.

### 5.6. Source Representation Effect Under a Fixed Backend Compressor

The proposed method should not be interpreted as a replacement for general-purpose entropy coders such as gzip or zstd. Instead, its role is to act as a geometry-aware source transformation that converts voxel occupancy into ordered contour-symbol streams plus positional metadata. The backend compressor is then applied to this transformed source. Therefore, the relevant question is not whether zstd contributes to the final compressed size; it clearly does. The relevant question is whether the proposed serialization produces a source representation that is substantially more compressible than alternative voxel representations under the same backend compressor.

The results support this interpretation. Throughout the main 40-class X-axis evaluation, the same zstd backend and compression level are applied to the proposed F4, 3OT, and F8 streams as well as to OCC1, BINVOX, and Octree BFS representations. If zstd alone were the dominant factor, these representations would be expected to behave similarly under the same backend. Instead, zstd-compressed chain-code streams, especially zstd + F8 from N = 32 onward, outperform zstd-compressed baseline representations. This indicates that the gain is not attributable to zstd alone, but to the fact that contour-based serialization changes the statistical structure of the source before backend compression.

Table 12 quantifies this source-transform view for the proposed streams. The raw serialized stream is the complete uncompressed chain-code representation passed to gzip or zstd, including contour-code symbols and the slice, component, hole, and positional metadata required for exact reconstruction. To avoid ambiguity, all stream-size values in Table 12 are reported in bits per voxel (bpv), using the same N^3 denominator as in the main compression evaluation. The zstd-compressed size is also reported in bpv, and the reduction column gives the percentage decrease from the raw serialized stream to the zstd-compressed stream.

Two observations follow. First, the raw streams quantify the cost of the contour-based representation before backend compression and show that the chain-code source transformation itself yields compact, structured streams. Second, zstd provides a substantial additional reduction because it can exploit the repeated directional transitions, local contour regularities, and regular metadata patterns exposed by that source transformation. Thus, zstd acts as a fixed final coding stage, while the methodological contribution lies in the representation supplied to that stage. The observed differences among F4, 3OT, and F8 under the same backend further show that the choice of chain-code representation matters: F8 is not merely benefiting from zstd, but provides a source stream whose directional structure is more effectively compressed at medium and high resolutions.

## 6. Discussion

The revised experiments show that the proposed contour-based serialization is resolution-dependent. At N = 8, the representation is not competitive with the best octree baseline because coarse grids provide little boundary detail, and metadata overhead is disproportionately large. From N = 16 onward, the proposed stream becomes favorable, and from N = 32 through N = 512, zstd + F8 is the strongest proposed configuration and also outperforms the best evaluated baseline. The object-level paired Wilcoxon tests in Table 5 further show that this behavior is statistically consistent over the evaluated population: the N = 8 disadvantage is significant, while the proposed configuration is significantly better than the strongest baseline from N = 16 through N = 512.

The shift from the earlier F4-centered interpretation to F8 is important. F4 and 3OT remain useful contour-coding instantiations, but the final 40-class evaluation shows that F8 better exposes the contextual and sequential structure exploited by zstd at medium and high resolutions. This interpretation is supported by the entropy analysis: 3OT dominates order-0 ideal code length because of its compact alphabet, whereas F8 dominates order-1 and order-2 ideal criteria.

The method should not be interpreted as a replacement for G-PCC in all point-cloud settings. G-PCC is a mature geometry codec designed for general point-cloud compression, including use cases outside binary voxel occupancy. The present contribution is narrower: a deterministic source serialization for exact binary occupancy of voxelized CAD-like objects. Within this scope, the best proposed stream outperforms G-PCC from N = 64 through N = 512, where G-PCC is the strongest baseline in the evaluated comparison.

The ablation results separate several design choices that were previously evaluated only jointly. Relative positional metadata reduces size compared with absolute positions, explicit separators add consistent overhead, and the FULL stream remains the most stable overall representation. Table 12 should be interpreted in the same representation-level sense: it does not assign the contribution to zstd alone; rather, it shows that chain-code serialization acts as a geometry-aware source transform. The same backend compressor is held fixed, and its different outcomes across F4, 3OT, F8, and the baseline representations indicate that the source stream supplied to zstd determines the achievable compression. The backend compressor is therefore not the source of novelty but a controlled final coding stage used to demonstrate that the proposed contour-based serialization exposes regularities that standard compressors can exploit more effectively than conventional voxel representations.

The computational analysis shows that runtime increases strongly with resolution, while differences among F4, 3OT, and F8 are smaller than the resolution effect. Memory measurements are also reported to make the resource cost explicit. This addresses the practical question of whether the source transformation is feasible beyond compressed-size comparisons.

The method has limitations. The main evaluation uses ModelNet40-derived CAD-like binary occupancy rather than outdoor LiDAR sequences or dynamic point clouds. Attributes and colors are discarded before occupancy coding. The evaluation is lossless, so lossy PCC metrics such as D1/D2 PSNR or BD-rate are outside this scope. Extreme-density LiDAR conditions and learned entropy coders are left for future work. The current results should therefore be interpreted as evidence for exact compression of voxelized object occupancy, not as a universal point-cloud compression claim. Additional future work should also compare the present pixel-center F4 and left-priority F8 tracers against crack-code F4 and alternative eight-neighbor F8 tracing variants under the same serialization and validation protocol.

## 7. Conclusions

This paper presented a lossless contour-based serialization for voxelized 3D objects. The method converts binary occupancy grids into slice-wise contour streams, separates foreground contours, hole contours, and positional metadata, and then applies conventional lossless compression.

The revised 40-class ModelNet40 evaluation shows that the method is resolution-dependent. It is not competitive with the best baseline at N = 8, but it outperforms the strongest evaluated baseline from N = 16 onward. The best proposed configuration is zstd + 3OT at N = 8 and N = 16, while zstd + F8 dominates from N = 32 through N = 512, with gains over the best baseline increasing to 84.37% at N = 512. The paired Wilcoxon analysis confirms that these bpv differences are statistically significant at the object level, with the expected negative result at N = 8 and significant advantages from N = 16 onward.

The entropy analysis explains this behavior: 3OT benefits from a compact alphabet under order-0 ideal coding, whereas F8 better captures contextual structure under order-1 and order-2 models. Ablations and the source-representation analysis further show that relative metadata, implicit contour termination, stream organization, and backend compression interact to produce the final compact representation. In particular, gzip and zstd are not methodological novelties; they are fixed backend stages that exploit the regular contour-symbol and metadata streams produced by the proposed chain-code serialization. Timing, memory, and validation results confirm exact reconstruction across all evaluated axes, resolutions, and contour coders and quantify resource costs.

These findings support contour-aware source serialization as an interpretable and reproducible path for exact binary voxel occupancy compression. Future work should evaluate outdoor LiDAR-like data, dynamic scenes, attributes, adaptive connectivity, learned entropy models, and alternative slice orderings, as well as crack-code variants and alternative eight-neighbor F8 tracing rules.

## Figures and Tables

**Table 1:** Representative state-of-the-art works related to contour-based voxel and point cloud geometry compression.

Work	Family	Main Idea	Relation to This Paper
Shannon [2]	Source coding	Entropy-limited compression framework for discrete sources.	Provides the information-theoretic basis for representation-dependent compressibility.
Freeman [3,4]	Contour coding	Directional chain codes for ordered digital contours.	Motivates the slice-wise contour serialization used here.
Žalik and collaborators [8,9,10]	2D binary chain-code compression	Compresses chain-coded binary shapes and masks using MTF/RLE, universal coding, and fast raster reconstruction.	Direct 2D binary-image compression lineage for the slice-wise contour streams used here.
Sánchez-Cruz and Rodríguez-Dagnino [5]; Sánchez-Cruz et al. [6]	3OT and chain-code efficiency	Three-orthogonal relative chain code and comparative efficiency analysis of F4, F8, VCC, and 3OT for binary objects.	Provides the specific 3OT genealogy and supports treating F4 and 3OT as alternative contour-coding instantiations.
Sánchez-Cruz et al. [7]; Repnik et al. [13]	3D/voxelized chain coding	Relative chain coding for 3D discrete curves and chain-code representation of voxelized solids.	Connects chain-code representations with three-dimensional and voxelized geometry, the closest prior line to this paper’s representation-level contribution.
Kim and Lee [11]	Voxel-surface coding	Pattern codes over 3 × 3 × 3 voxel neighborhoods.	Closest classical lossless voxel-surface reference: boundary-focused but not slice-chain based.
Kwon et al. [12]	Progressive voxel coding	Pyramidal decomposition and boundary-oriented arithmetic coding.	Shows that multiresolution and boundary structure are useful in binary voxel compression.
Schnabel and Klein [14]	Octree PCC	Lossless octree point-cloud compression with geometric prediction.	Canonical non-contour sparse-geometry baseline.
Graziosi et al.; ISO/IEC 23090-9 [16,17]	Standardized PCC	MPEG V-PCC/G-PCC standardization and geometry-based PCC.	Justifies G-PCC as a strong adjacent benchmark.
OctSqueeze [18]	Learned the octree model	Tree-structured entropy model for LiDAR point clouds.	Represents the learned octree-probability branch of PCC.
VoxelContext-Net [19]	Learned octree/voxel context	Local voxel context for octree symbols in static/dynamic point clouds.	Shows the value of voxel context but remains octree-centered.
VoxelDNN/MSVoxelDNN [20,21]	Learned lossless geometry	Autoregressive and multiscale voxel probability models.	Strong learned lossless PCC references; highlight complexity/serial-dependence issues.
SparsePCGC [22]	Sparse tensor PCC	Multiscale sparse tensor occupancy-probability modeling.	Recently learned sparse-voxel direction, complementary to deterministic serialization.
Nguyen and Kaup [23]	Learned geometry + attributes	Sparse tensor conditional probability model for lossless geometry and color.	Modern benchmark showing the strength of learned context modeling.

**Table 2:** Timeline of major developments relevant to contour-based voxel compression.

Year	Milestone
1948	Shannon establishes the mathematical source-coding framework for communication and compression [2].
1961/1974	Freeman introduces and surveys chain-code representations for digital contours [3,4].
2002	Pattern-code representation is proposed for lossless 3D binary voxel-surface compression [11].
2004	Progressive binary voxel-model coding is introduced using pyramidal decomposition [12].
2005/2007	Sánchez-Cruz and collaborators introduce and evaluate 3OT as a relative three-symbol chain code for bilevel/binary object contours [5,6].
2006	Octree-based point cloud compression becomes a key sparse-geometry baseline [14].
2014	A relative chain code for 3D discrete curves is proposed, extending the chain-code lineage toward voxel-based three-dimensional geometry [7].
2020	MPEG PCC standardization literature consolidates V-PCC and G-PCC reference frameworks [17].
2020	OctSqueeze introduces learned octree entropy modeling for LiDAR point clouds [18].
2021	VoxelContext-Net and VoxelDNN/MSVoxelDNN advance learned voxel/octree context modeling [19,20,21].
2023	ISO/IEC 23090-9 is published as the G-PCC international standard [16].
2023	Sparse tensor and conditional-probability models extend learned lossless point-cloud coding [22,23].
2025	A chain-code representation for voxelized solids is proposed, reporting compact binary storage, lower entropy, and improved gzip/bzip2 compressibility for generated token streams [13].
This work	Contour-aware slice serialization is evaluated as a deterministic source representation for exact binary voxel occupancy.

**Table 3:** Final evaluated representation-level methods and baselines.

Method	Category	Representation	Description	Final Role
chaincode_F4_text	Proposed	Contour stream using F4	Absolute four-neighbor orthogonal chain-code serialization.	Included in the main 40-class X-axis evaluation.
chaincode_3OT_text	Proposed	Contour stream using 3OT	Relative three-symbol orthogonal chain-code serialization.	Best proposed stream at N = 8 and N = 16 with zstd.
chaincode_F8_text	Proposed	Contour stream using F8	Eight-neighbor chain-code serialization.	Best proposed stream from N = 32 to N = 512 with zstd.
OCC1	Baseline	One-bit occupancy stream	Simple raw binary occupancy layout.	Final baseline.
BINVOX	Baseline	Voxel file format	Voxel-storage baseline compressed with gzip/zstd.	Final baseline.
octree_mask_bfs	Baseline	Breadth-first octree mask	Canonical pointerless octree-occupancy mask.	Final baseline.
G-PCC/TMC13	Baseline	Geometry-only point-cloud codec	Occupied voxel centers encoded as a point cloud using TMC13 geometry-only mode.	Final baseline.
custom_svo_leaf2	Generated but excluded	Sparse voxel octree variant	Generated during experimentation.	Excluded due to redundancy with octree_mask_bfs.

**Table 4:** Main 40-class X-axis compression results by resolution.

N	Best Proposed	Proposed bpv	Best Baseline	Baseline bpv	Gain	Rank
8	zstd + 3OT	0.831028	zstd + Octree BFS	0.680196	−22.17%	4/13
16	zstd + 3OT	0.202745	zstd + OCC1	0.256410	+20.93%	1/13
32	zstd + F8	0.058895	zstd + OCC1	0.116222	+49.33%	1/13
64	zstd + F8	0.020319	G-PCC	0.055811	+63.59%	1/13
128	zstd + F8	0.007498	G-PCC	0.026937	+72.16%	1/13
256	zstd + F8	0.002688	G-PCC	0.012906	+79.17%	1/13
512	zstd + F8	0.000949	G-PCC	0.006072	+84.37%	1/13

**Table 5:** Paired Wilcoxon signed-rank tests for the main 40-class X-axis bpv comparisons.

N	Comparison	n	Median Diff bpv	*p*-Value	Interpretation
8	zstd + 3OT vs. zstd + Octree BFS	3983	−0.156250	<0.001	Baseline better
16	zstd + 3OT vs. zstd + OCC1	3983	0.025391	<0.001	Proposed better
32	zstd + F8 vs. zstd + OCC1	3983	0.036377	<0.001	Proposed better
64	zstd + F8 vs. G-PCC	3983	0.027161	<0.001	Proposed better
128	zstd + F8 vs. G-PCC	3983	0.014725	<0.001	Proposed better
256	zstd + F8 vs. G-PCC	3983	0.007730	<0.001	Proposed better
512	zstd + F8 vs. G-PCC	3983	0.003875	<0.001	Proposed better

**Table 6:** Class-wise wins against the best non-chain-code baseline.

N	Wins	Losses	Win Rate	Mean Delta bpv	Strongest Gain	Largest Loss/Smallest Gain
8	3	37	7.5%	−0.165480	dresser (0.0884)	plant (−0.4391)
16	30	10	75.0%	0.051992	night_stand (0.2696)	plant (−0.1424)
32	36	4	90.0%	0.054301	night_stand (0.1936)	plant (−0.0562)
64	38	2	95.0%	0.033462	glass_box (0.1115)	plant (−0.0214)
128	39	1	97.5%	0.018735	glass_box (0.0580)	plant (−0.0060)
256	39	1	97.5%	0.009882	glass_box (0.0293)	plant (−0.0002)
512	40	0	100.0%	0.004968	glass_box (0.0139)	guitar (0.0002)

**Table 7:** Overall entropy winner counts for F4, 3OT, and F8.

Criterion	F4 Wins	3OT Wins	F8 Wins	Ties	Dominant Method	Interpretation
Symbol length	13	0	25,761	2107	F8	F8 most often produces the shortest symbol streams.
Raw chars	0	0	25,813	2068	F8	F8 also dominates raw character counts.
Ideal order-0 bits	175	27,424	111	171	3OT	3OT benefits from its compact alphabet.
Ideal order-1 bits	39	8968	17,988	886	F8	F8 better captures first-order context.
Ideal order-2 bits	46	5643	21,072	1120	F8	F8 better captures second-order context.

**Table 8:** Ablation summary under zstd for the four-class deep-analysis subset.

Method	Variant	Mean bpv	Median bpv	Mean bpo	Overhead vs. FULL	Files
F4	FULL	0.182119	0.162876	1.802786	0.00%	8400
F4	ABS	0.182952	0.162850	1.814152	+1.59%	8400
F4	SEP	0.196795	0.176583	1.930867	+3.63%	8400
F4	INT	0.182828	0.163434	1.806519	+0.04%	8400
3OT	FULL	0.180345	0.165142	1.810638	0.00%	8400
3OT	ABS	0.181115	0.165618	1.821308	+1.59%	8400
3OT	SEP	0.196466	0.181523	1.956256	+4.72%	8400
3OT	INT	0.180726	0.165625	1.812432	−0.15%	8400
F8	FULL	0.182082	0.162750	1.796743	0.00%	8400
F8	ABS	0.182729	0.162475	1.804908	+0.96%	8400
F8	SEP	0.196861	0.178252	1.925945	+3.49%	8400
F8	INT	0.182686	0.163199	1.799857	−0.03%	8400

**Table 9:** Encoding and decoding wall-clock time by resolution, mean ± 95% CI in milliseconds.

N	Enc F4	Enc 3OT	Enc F8	Fast Enc	Dec F4	Dec 3OT	Dec F8	Fast Dec
8	2.6 ± 0.2	2.0 ± 0.2	2.0 ± 0.2	F8	5.7 ± 0.1	6.0 ± 0.1	5.7 ± 0.1	F4
16	3.6 ± 0.2	3.2 ± 0.2	3.1 ± 0.2	F8	6.7 ± 0.1	7.0 ± 0.1	6.7 ± 0.1	F8
32	5.5 ± 0.2	5.5 ± 0.2	5.9 ± 0.2	F4	9.0 ± 0.1	9.0 ± 0.1	8.5 ± 0.1	F8
64	18.3 ± 0.4	18.3 ± 0.4	19.9 ± 0.4	3OT	18.7 ± 0.3	18.7 ± 0.3	17.7 ± 0.3	F8
128	104.7 ± 2.3	104.2 ± 2.2	110.1 ± 2.4	3OT	69.3 ± 1.8	69.9 ± 1.8	68.6 ± 1.8	F8
256	697.6 ± 16.6	697.1 ± 16.7	726.0 ± 17.3	3OT	430.2 ± 14.0	431.6 ± 14.0	430.9 ± 14.2	F4
512	5221.4 ± 133.1	5249.0 ± 135.3	5410.1 ± 138.0	F4	3257.8 ± 115.1	3263.5 ± 115.1	3264.9 ± 114.9	F4

**Table 10:** Overall timing and memory summary across X-axis resolutions.

Method	Enc Mean	Enc Median	Enc p95	Dec Mean	Dec Median	Dec p95	Enc Peak MB	Dec Peak MB
F4	864.821	19.660	5732.787	542.487	15.934	3271.085	268.75	141.22
3OT	868.454	17.250	5761.394	543.681	15.801	3277.735	283.22	137.78
F8	896.732	18.647	5953.775	543.265	14.550	3271.539	283.42	139.93

**Table 11:** Coverage and exact decoding validation summary.

Item	Value
Encoding records	250,929
Decoding records	250,929
Classes	40
Distinct objects	3983
Resolutions	8, 16, 32, 64, 128, 256, 512
Methods	F4, 3OT, F8
Axes	X, Y, Z
Exact decoding OK	250,929
Flagged exceptions	0 (seven earlier Z-axis exceptions corrected and re-validated; see Section 5.5)
Main 40-class X-axis failures	0

**Table 12:** Raw serialized and zstd-compressed contour-stream sizes for F4, 3OT, and F8 over the 40-class X-axis evaluation. Size values are reported in bits per voxel (bpv), and zstd reduction is reported as a percentage.

N	Method	Raw Serialized Size (bpv)	zstd-Compressed Size (bpv)	zstd Reduction (%)
8	F4	2.155473	0.845931	60.8
8	3OT	2.474199	0.831028	66.4
8	F8	1.977514	0.848583	57.1
16	F4	1.146491	0.203283	82.3
16	3OT	1.251108	0.202745	83.8
16	F8	1.047672	0.202809	80.6
32	F4	0.640748	0.059517	90.7
32	3OT	0.676070	0.060974	91.0
32	F8	0.587309	0.058895	90.0
64	F4	0.370720	0.020698	94.4
64	3OT	0.383822	0.021286	94.5
64	F8	0.341533	0.020319	94.1
128	F4	0.216407	0.007647	96.5
128	3OT	0.221427	0.007780	96.5
128	F8	0.200323	0.007498	96.3
256	F4	0.118755	0.002729	97.7
256	3OT	0.120410	0.002750	97.7
256	F8	0.110288	0.002688	97.6
512	F4	0.061969	0.000967	98.4
512	3OT	0.062463	0.000973	98.4
512	F8	0.057608	0.000949	98.4

## Data Availability

The original ModelNet40 dataset is publicly available from its corresponding source. The final aggregated compression, entropy, ablation, timing, memory, GC, and validation results supporting the findings of this revised manuscript are provided in the Supplementary Files listed above. The derived voxelized files, chain-code streams, scripts, and intermediate data generated during the study are available from the corresponding author upon reasonable request, subject to storage constraints and dataset redistribution rules.

## Supplementary Materials

The following supporting information can be downloaded at: https://www.mdpi.com/article/10.3390/e28070774/s1, S1_ModelNet40_XAxis_Compression_Summary.xlsx: Main 40-class X-axis compression workbook, including per-resolution summaries, class-wise rankings, wins/losses, and figure data for the proposed streams and baselines. S2_ModelNet40_XAxis_Compression_RecordTable.csv: Filtered long-format object-level compression record table with occupied-voxel counts filled for the main 40-class X-axis evaluation. S3_ChainCode_Entropy_Analysis_F4_3OT_F8.xlsx: Order-0, order-1, and order-2 entropy analysis for F4, 3OT, and F8 contour-symbol streams over the 40-class X-axis evaluation. S4_FourClass_XYZ_Ablation_Study.xlsx: Four-class X/Y/Z ablation workbook for FULL, ABS, SEP, and INT variants using airplane, bookshelf, chair, and vase. S5_EncodingDecoding_Timing_Validation_Analysis.xlsx: Encoding/decoding timing, memory, garbage-collection, and exact-decoding validation workbook for the chain-code pipeline. S6_Paired_Wilcoxon_BPV_Statistics.zip: Paired Wilcoxon signed-rank statistics for the main per-object bpv comparisons, including main tests, object-level differences, and pairwise comparison matrices. S7_SourceRepresentation_BackendCompressor_Analysis.zip: Raw serialized stream versus backend-compressed size analysis used to quantify the source-representation effect under fixed gzip/zstd settings. S8_Reproducibility_Protocol.docx: Reproducibility protocol documenting software versions, compressor levels, voxelization, G-PCC/TMC13 settings, hardware context, and execution notes.

## References

[1] Sangaiah A.K., Anandakrishnan J., Kumar S., Bian G.-B., AlQahtani S.A., Draheim D. (2026). Point-KAN: Leveraging Trustworthy AI for Reliable 3-D Point Cloud Completion with Kolmogorov-Arnold Networks for 6G-IoT Applications. IEEE Internet Things J..

[2] Shannon C.E. (1948). A Mathematical Theory of Communication. Bell Syst. Tech. J..

[3] Freeman H. (1961). On the Encoding of Arbitrary Geometric Configurations. IRE Trans. Electron. Comput..

[4] Freeman H. (1974). Computer Processing of Line-Drawing Images. ACM Comput. Surv..

[5] Sánchez-Cruz H., Rodríguez-Dagnino R.M. (2005). Compressing Bilevel Images by Means of a Three-Bit Chain Code. Opt. Eng..

[6] Sánchez-Cruz H., Bribiesca E., Rodríguez-Dagnino R.M. (2007). Efficiency of Chain Codes to Represent Binary Objects. Pattern Recognit..

[7] Sánchez-Cruz H., López-Valdez H.H., Cuevas F.J. (2014). A New Relative Chain Code in 3D. Pattern Recognit..

[8] Žalik B., Lukač N. (2014). Chain Code Lossless Compression Using Move-to-Front Transform and Adaptive Run-Length Encoding. Signal Process. Image Commun..

[9] Žalik B., Mongus D., Lukač N. (2015). A Universal Chain Code Compression Method. J. Vis. Commun. Image Represent..

[10] Strnad D., Žlaus D., Nerat A., Žalik B. (2025). Efficient Compressed Storage and Fast Reconstruction of Large Binary Images Using Chain Codes. Multimed. Tools Appl..

[11] Kim C.-S., Lee S.-U. (2002). Compact Encoding of 3-D Voxel Surfaces Based on Pattern Code Representation. IEEE Trans. Image Process..

[12] Kwon M., Kim C.-S., Lee K.M., Lee S.-U. (2004). Progressive Encoding of Binary Voxel Models Using Pyramidal Decomposition. J. Vis. Commun. Image Represent..

[13] Repnik B., Váša L., Žalik B. (2025). An Algorithm for Voxelised Solids Representation Using Chain Codes. Signal Process. Image Commun..

[14] Schnabel R., Klein R. Octree-Based Point-Cloud Compression. Proceedings of the Eurographics Symposium on Point-Based Graphics.

[15] Laine S., Karras T. Efficient Sparse Voxel Octrees. Proceedings of the ACM SIGGRAPH Symposium on Interactive 3D Graphics and Games.

[16] (2023). Information Technology-Coded Representation of Immersive Media-Part 9: Geometry-Based Point Cloud Compression.

[17] Graziosi D., Nakagami O., Kuma S., Zaghetto A., Suzuki T., Tabatabai A. (2020). An Overview of Ongoing Point Cloud Compression Standardization Activities: Video-Based (V-PCC) and Geometry-Based (G-PCC). APSIPA Trans. Signal Inf. Process..

[18] Huang L., Wang S., Wong K., Liu J., Urtasun R. OctSqueeze: Octree-Structured Entropy Model for LiDAR Compression. Proceedings of the IEEE/CVF Conference on Computer Vision and Pattern Recognition (CVPR).

[19] Que Z., Lu G., Xu D. VoxelContext-Net: An Octree Based Framework for Point Cloud Compression. Proceedings of the IEEE/CVF Conference on Computer Vision and Pattern Recognition (CVPR).

[20] Nguyen D.T., Quach M., Valenzise G., Duhamel P. Learning-Based Lossless Compression of 3D Point Cloud Geometry. Proceedings of the IEEE International Conference on Acoustics, Speech and Signal Processing (ICASSP).

[21] Nguyen D.T., Quach M., Valenzise G., Duhamel P. Multiscale Deep Context Modeling for Lossless Point Cloud Geometry Compression. Proceedings of the IEEE International Conference on Multimedia and Expo Workshops (ICMEW).

[22] Wang J., Ding D., Li Z., Feng X., Cao C., Ma Z. (2023). Sparse Tensor-Based Multiscale Representation for Point Cloud Geometry Compression. IEEE Trans. Pattern Anal. Mach. Intell..

[23] Nguyen D.T., Kaup A. (2023). Lossless Point Cloud Geometry and Attribute Compression Using a Learned Conditional Probability Model. IEEE Trans. Circuits Syst. Video Technol..

[24] Tian D., Ochimizu H., Feng C., Cohen R., Vetro A. Geometric Distortion Metrics for Point Cloud Compression. Proceedings of the IEEE International Conference on Image Processing (ICIP).

[25] Wu Z., Song S., Khosla A., Yu F., Zhang L., Tang X., Xiao J. 3D ShapeNets: A Deep Representation for Volumetric Shapes. Proceedings of the IEEE Conference on Computer Vision and Pattern Recognition (CVPR).

[26] Eisenwave obj2voxel: Convert OBJ and STL Files to Voxels, with Support for Textures. https://github.com/eisenwave/obj2voxel.

[27] Deutsch P. (1996). RFC 1951: DEFLATE Compressed Data Format Specification version 1.3.

[28] Collet Y., Kucherawy M. (2021). RFC 8878: Zstandard Compression and the Application/zstd Media Type.

[29] Min P. binvox: 3D Mesh Voxelizer and BINVOX File Format. https://www.patrickmin.com/binvox/.

[30] MPEGGroup mpeg-pcc-tmc13: Geometry Based Point Cloud Compression Test Model. https://github.com/MPEGGroup/mpeg-pcc-tmc13.

