# Supplementary material for: Contour-Based Chain-Code Serialization for Lossless Compression of Voxelized 3D Objects
Source: Entropy (Basel). 2026 Jul 8;28(7):774. doi: 10.3390/e28070774 (PMC13408810; doi:10.3390/e28070774)
Supplement: Supplementary file 1 [file entropy-28-00774-s001.zip › S8_Reproducibility_Protocol.pdf]

# Reproducibility Protocol

Contour-Based Chain-Code Serialization for Lossless Compression of Voxelized 3D Objects

## 1. Scope

This protocol documents the reproducibility conditions for the revised manuscript. The evaluation concerns lossless compression of binary voxel occupancy derived from ModelNet40 objects. The main experiment uses 40 classes along the X slicing axis. A complementary deep-analysis subset uses airplane, bookshelf, chair, and vase across X, Y, and Z axes for exact reconstruction, ablations, timing, memory, and validation.

## 2. Dataset and evaluation scope

| Item | Value |
|---|---|
| Dataset | ModelNet40-derived voxelized objects |
| Object count | 3983 objects |
| Classes | 40 classes; 38 classes with 100 objects, bowl with 84, cup with 99 |
| Resolutions | N = 8, 16, 32, 64, 128, 256, 512 |
| Main slicing axis | X axis |
| Deep-analysis subset | airplane, bookshelf, chair, vase across X/Y/Z |

## 3. Representations and baselines

| Category | Specification |
|---|---|
| Proposed | F4, 3OT, and F8 contour-code streams with slice/component/hole/position metadata |
| Baselines | OCC1, BINVOX, Octree BFS (octree_mask_bfs), geometry-only G-PCC/TMC13 |
| Generated but excluded | custom_svo_leaf2, excluded from final analysis as redundant with octree_mask_bfs |
| Compressor settings | gzip/DEFLATE level 9; zstd level 19 |

## 4. Software and toolchain

| Component | Version or setting |
|---|---|
| Python | 3.12.10 |
| numpy | 1.26.4 |
| pandas | 3.0.0 |
| trimesh | 3.23.5 |
| openpyxl | 3.1.5 |
| zstandard | 0.25.0 |
| Voxelization | Obj2Voxel.exe / VoxelList-RGB; OBJ input; selected -r N resolution |
| G-PCC / TMC13 | tmc3 78ca512+dirty; Windows x64 Release; Visual Studio 17 2022; MSVC v143 |

## 5. G-PCC command

Geometry-only G-PCC was executed with attribute coding disabled. The command used was:

```
tmc3.exe --mode=0 --uncompressedDataPath=<input.ply> --compressedStreamPath=<output.gpcc>
--disableAttributeCoding=1
```

## 6. Metrics

The principal publication metric is bits per voxel (bpv), computed as 8 × compressed_bytes / N^3. Bits per occupied voxel (bpo) is computed as 8 × compressed_bytes / occupied_voxels. For the source-representation-under-fixed-backend analysis, raw_bpv is computed from original_bytes before gzip/zstd, while compressed_bpv is computed from compressed_bytes after backend compression.

## 7. Determinism and reproducibility

After voxelization, the proposed encoding pipeline is deterministic for a fixed input object, target resolution, slicing axis, contour coder, serialization rule, and compressor setting. No training stage, stochastic model fitting, randomized sampling, or k-fold validation is used because the proposed representation is not a learned model.

## 8. Timing hardware

Timing values for the chain-code encoder and decoder were measured on a conventional Windows 10 Home machine with an Intel Core i7-10870H CPU @ 2.20 GHz, 32 GB RAM, and 64-bit x64 architecture. Large-scale compression experiments were executed incrementally across multiple machines; timing values are therefore reported only for the chain-code encoding/decoding analysis.

## 9. Validation and known exceptions

The main 40-class X-axis compression evaluation has zero exact-decoding failures. Across the broader X/Y/Z timing and validation dataset, 250922 of 250929 decoding records were exact. The seven flagged failures correspond to flower_pot cases on the Z axis. These cases do not affect the main 40-class X-axis compression evaluation.

## 10. Supplementary files

The supplementary package includes the main 40-class X-axis compression workbook, the entropy workbook, the four-class ablation workbook, the timing/validation workbook, the Wilcoxon bpv package, the source-representation/backend-compressor analysis package, and this reproducibility protocol.
